# Early childhood caries prevalence and associated risk factors among Saudi preschool children in Riyadh

**DOI:** 10.1016/j.sdentj.2021.04.003

**Published:** 2021-04-21

**Authors:** Lujane K. AlMarshad, Amjad H. Wyne, Asma M. AlJobair

**Affiliations:** a Department of Pediatric Dentistry and Orthodontics, College of Dentistry, King Saud University, Riyadh, Saudi Arabia; b Pediatric Dentistry Department, CMH Lahore Medical College & Institute of Dentistry, Lahore, Pakistan

**Keywords:** Early childhood caries, Preschool children, Prevalence, Caries risk factors, Saudi Arabia

## Abstract

**Aim:**

To determine the prevalence of early childhood caries (ECC) and investigate the effect of associated risk factors on ECC prevalence in preschool children in Riyadh, Saudi Arabia.

**Methods:**

This cross-sectional study included Saudi preschoolers aged 36–71 months. Parents/guardians completed a structured, self-administered questionnaire assessing sociodemographics; medical, dental, and dietary history; and oral hygiene practices. Children were orally examined for dental caries, oral hygiene, and plaque deposition.

**Results:**

A total of 383 children were examined. ECC prevalence was 72.6%, with a mean decayed, missing, and filled teeth (dmft) score of 4.13 (±3.99) and a mean decayed, missing, and filled surfaces (dmfs) score of 7.0 (±9.1). Children from schools in northern Riyadh and those of fathers in professional jobs were less likely to have ECC [(OR: 0.203; 95% CI: 0.082–0.503)] and [(OR: 0.472; 95% CI: 0.256–0.871)], respectively. Children with a nocturnal feeding history and poor oral hygiene were more likely to have ECC [(OR: 2.281; 95% CI: 1.143–4.553)] and [(OR: 5.523; 95% CI: 2.269–13.441)], respectively.

**Conclusions:**

The prevalence of ECC in preschool children in Riyadh is high and affected by parental socioeconomic factors, infant feeding practices, and children’s oral hygiene status.

## 1 Introduction

Early childhood caries (ECC) has been defined as “The presence of one or more decayed, missing, or filled tooth surfaces in any primary tooth in a child 71 months of age or younger” (American Academy of Pediatric Dentistry, 2017).

Only 45% of United Nations member countries have data on ECC as defined by the American Academy of Pediatric Dentistry (El Tantawi et al., 2018). In the Kingdom of Saudi Arabia, available caries data are mainly from uncoordinated independent studies; there are no nationwide surveys on ECC, probably owing to logistic issues and difficulty in systematization (Al-Ansari et al., 2019). However, studies of children younger than 6 years in various local populations in Saudi Arabia have reported an ECC prevalence from 62% to 84%, with a mean number of decayed, missing, and filled teeth (dmft) scores of 3.0 to 7.1 (Al Agili, 2013). A study in Riyadh, the Saudi Arabian capital, reported an ECC prevalence of 74.8% with a mean dmft score of 6.1 (Wyne, 2008). The most recently reported ECC prevalence in preschool children in Riyadh was 69%, with a mean dmft score of 3.4, indicating a decline and changes in factors associated with ECC (Al-Meedani and Al-Dlaigan, 2016).

ECC is a multifactorial risk disease. In an ECC prediction model study among Saudi preschoolers in Riyadh, Al Ghanim et al. (1998) identified poor oral hygiene and feeding/dietary habits as the main risk factors. However, more than a decade later, ECC risk factors seem to have changed; Al-Meedani and Al-Dlaigan (2016) reported that ECC is associated with socioeconomic factors in the same population.

Owing to these dynamic changes in ECC prevalence and risk factors, more research is needed on Saudi preschoolers to customize dental services and effectively direct preventive measures. Therefore, this study aimed to determine the prevalence of ECC and investigate the associated risk factors in Riyadh preschoolers.

## 2 Materials and methods

### 2.1 Ethical considerations

Institutional review board ethical approval was obtained (E-17–2230) and the study was registered with KSU College of Dentistry Research Centre (PR-0064). Informed consent was obtained from all parents of the selected children.

### 2.2 Study design and sample

A cross-sectional study was conducted in 2018–2019 in Riyadh, using stratified random sampling. A list of all preschools was obtained from the Ministry of Education. Using a randomization website (https://www.randomizer.org/), two preschools (one public, one private) were selected from each administrative region (central, northern, southern, eastern, and western) of Riyadh city. A letter was sent to principals of each selected school from the Ministry of Education informing them of the study. According to available data from the previous academic year (2017–2018), there were 45,687 Saudi preschool children in Riyadh city, enrolled in 633 preschools. Using a confidence level of 95% and a margin of error of 5%, the sample size was calculated as 381 children. The inclusion criteria were healthy Saudi children with no chronic illnesses, no syndromes, no continuous use of medication, and aged 36–71 months.

### 2.3 Questionnaire

A questionnaire comprising items on sociodemographic data, medical history, dental history, infant feeding history, dietary habits, and oral hygiene practice was constructed and translated into Arabic and then back to English to ensure accuracy. Two dental faculty members confirmed face validity. The questionnaire test–retest reliability was assessed on two different days with 25 parents not included in the main study. The questionnaires were then distributed to all children in each selected school with a covering letter for informed consent. Questionnaires were completed by parents and collected 2 weeks later. Participation was voluntary, and responses remained anonymous (serial numbers replaced names). After parents signed the informed consent forms, the principal investigator reviewed the questionnaires for appropriateness and children who fulfilled the inclusion criteria were dentally examined.

### 2.4 Clinical examination

The principal investigator performed an intraoral examination in the preschools, using a portable light, disposable examination instruments (mouth mirror, explorer, and cotton pliers), and personal protective barriers. The child was seated on a portable chair. World Health Organization criteria were used to diagnose dental caries (World Health Organization, 2013). Decayed, missing, and filled teeth/surfaces (dmft/dmfs) scores were calculated for each child. Oral hygiene was assessed using the James et al. (1960) Oral Hygiene Index (OHI). Plaque deposition was recorded using the Loe Plaque index (PI) (Loe, 1967). Data were recorded on a form specially designed for the study. The intra-examiner reliability for dmfs scores was confirmed by re-examining 10% of randomly selected children after 2 weeks. Cronbach’s alpha was 0.961.

### 2.5 Statistical analysis

The Statistical Package for the Social Sciences (SPSS) version 26.0 (SPSS Inc., Chicago, IL, USA) was used for data analysis. Some of the original variable categories were combined to facilitate statistical analyses; more than half of the mothers did not work, so mother’s occupation was grouped into working or non-working. Father's occupation was grouped into: administrative (public, private sector) or professional (Doctor, engineer, technician…). Parents’ education was categorized as low education (illiterate, elementary, intermediate, and secondary schooling) and high education (bachelor and postgraduate). Frequency of breast- or bottle-feeding during infancy was categorized as < 7/day and ≥ 7/day. PI was divided into two groups (low: ≤1, high: >1). Various frequencies were generated. Univariate analyses were conducted using the chi-square test to determine associations between each factor and ECC. The significance level was p < 0.05. To control for covariates, variables with p-values < 0.1 were included in a multiple logistic regression model.

## 3 Results

Of the 424 children whose parents completed the questionnaire, 41 were excluded as they did not meet the inclusion criteria. Hence, 383 children were examined; the mean age was 60.9 (±8.06) months and more than half (54.8%) were girls. The prevalence of ECC was 72.6%; the mean dmft score was 4.13 (±3.99) and the mean dmfs score was 7.0 (±9.1).

Table 1 shows demographic information and the association between ECC and sociodemographic and socioeconomic variables. There was a trend (p = 0.085) toward higher prevalence of ECC among older children compared with younger children. There was a significant association between ECC prevalence and preschool area: children from schools in northern Riyadh had the lowest ECC prevalence [(60.3%); p = 0.042]. Additionally, children of fathers with professional jobs had significantly lower ECC prevalence [(68.5%); p = 0.049].

**Table 1:** Association of ECC with sociodemographic and socioeconomic variables.

Factor		No. (%)	Caries grouping		p-value
			Caries free (%)	ECC (%)	
Age of child	36–47 months	26 (6.8)	12 (46.1)	14 (53.9)	0.085
	48–59 months	102 (26.6)	27 (26.5)	75 (73.5)	
	60–71 months	255 (66.6)	66 (25.9)	189 (74.1)	
Gender of child	Girls	210 (54.8)	57 (27.1)	153 (72.9)	0.895
	Boys	173 (45.2)	48 (27.7)	125 (72.3)	
Preschool type	Public	214 (55.9)	52 (24.3)	162 (75.7)	0.124
	Private	169 (44.1)	53 (31.4)	116 (68.6)	
Preschool area	East	49 (12.8)	11 (22.4)	38 (77.6)	0.042
	North	73 (19.1)	29 (39.7)	44 (60.3)	
	South	109 (28.5)	33 (30.3)	76 (69.7)	
	West	56 (14.6)	13 (23.2)	43(76.8)	
	Central	96 (25)	19 (19.8)	77 (80.2)	
Mother’s education	High school or below	121 (31.6)	33 (27.3)	88 (72.7)	0.914
	College or above	258 (67.4)	69 (26.7)	189 (73.3)	
Father’s education	High school or below	126 (33.2)	34 (27)	92 (73)	0.923
	College or above	255 (66.6)	70 (27.5)	185 (72.5)	
Mother’s occupation	Working	177 (46.2)	47 (26.6)	130 (73.4)	0.726
	Non-working	206 (53.8)	58 (28.2)	148 (71.8)	
Father’s occupation	Professional	216 (56.4)	68 (31.5)	148 (68.5)	0.049
	Administrative	167 (43.6)	37 (22.2)	130 (77.8)	
Birth order of child	First	96 (25)	28 (29.2)	68 (70.8)	0.102
	Middle	133 (34.7)	43 (32.3)	90 (67.7)	
	Last	150 (39.2)	32 (21.3)	118 (78.7)	
Number of children in family	1–2	128 (33.4)	38 (29.7)	90 (70.3)	0.736
	3–5	217 (56.6)	56 (25.8)	161 (74.2)	
	≥6	33 (8.6)	9 (27.3)	24 (72.7)	

ECC: early childhood caries.

Table 2 shows the association of ECC with infant feeding and dietary variables. A significantly higher ECC prevalence was found in children who were bottle/breast-fed more frequently as infants [(80.5%); p = 0.031]. Children with nocturnal feeding history had significantly higher ECC prevalence [(77.2%); p = 0.001]. Additionally, higher ECC prevalence was found in children who consumed sugary/acidic drinks while sleeping [(79.4%); p = 0.026). However, there was a trend toward lower ECC prevalence among children who consumed water day and night [(65.1%); p = 0.082]. Children who consumed sweets and soft drinks between meals more than once daily had the highest caries prevalence [(82.9%); p = 0.031].

**Table 2:** Association between ECC and history of infant feeding/dietary variables.

Factor		No. (%)	Caries grouping		p-value
			Caries free (%)	ECC (%)	
History of infant feeding					
Age stopped bottle/breast-feeding	<12 months	102 (26.6)	29 (28.4)	73 (71.6)	0.650
	12–18 months	110 (28.7)	26 (23.6)	84 (76.4)	
	>18 months	163 (42.6)	46 (28.2)	117 (71.8)	
Daily feeding frequency	<7/day	230 (60)	69 (30)	161 (70)	0.031
	≥7/day	128 (33.4)	25 (19.5)	103 (80.5)	
Nocturnal feeding (breast/bottle milk)	Yes	302 (78.9)	69 (22.8)	233 (77.2)	0.001
	No	81 (21.1)	36 (44.4)	45 (55.6)	
Sugary drinks in bottle (day)	Yes	189 (49.3)	49 (25.9)	140 (74.1)	0.519
	No	194 (50.7)	56 (28.9)	138 (71.1)	
Sugary drinks in bottle (night)	Yes	136 (35.5)	28 (20.6)	108 (79.4)	0.026
	No	247 (64.5)	77 (31.2)	170 (68.8)	
Water in bottle (day/night)	Yes	83 (21.7)	29 (34.9)	54 (65.1)	0.082
	No	300 (78.3)	76 (25.3)	224 (74.7)	
Current dietary habits					
Addition of sugar to meals	Yes	166 (43.3)	48 (29)	118 (71)	0.704
	No	210 (54.8)	57 (27.1)	153 (72.9)	
Consumption of sweets and soft drinks between meals	No	37 (9.7)	17 (45.9)	20 (54.1)	0.031
	Not daily	226 (59)	59 (26.1)	167 (73.9)	
	1/day	75 (19.6)	22 (29.3)	53 (70.7)	
	>1/day	41 (10.7)	7 (17.1)	34 (82.9)	

ECC: early childhood caries.

Table 3 shows the association of ECC with dental history and oral health-related variables. A higher prevalence of ECC was found in children who had visited a dentist [(84.9%); p < 0.0001]. Children whose dental visit was because of a dental problem had significantly higher ECC prevalence [(94.4%); p = 0.002]. The prevalence of ECC was higher in children with no/irregular brushing [(77.9%); p = 0.044]. There was also a strong association between ECC occurrence and higher PI [(77.0%); p = 0.004]. Similarly, there was a strong association between ECC occurrence and poor oral hygiene status [(88.1%); p < 0.0001].

**Table 3:** Association between ECC and dental history/oral health-related variables.

Factor		No. (%)	Caries grouping		p-value
			Caries free (%)	ECC (%)	
Dental history					
History of dental visits	Yes	152 (39.7)	23 (15.1)	129 (84.9)	<0.0001
	No	231 (60.3)	82 (35.5)	149 (64.5)	
Reason for first dental visit	Checkup	81 (21.1)	19 (23.5)	62 (76.5)	0.002
	Dental problem	71 (18.5)	4 (5.6)	67 (94.4)	
Age of first dental visit	≤1 year	32 (8.4)	4 (12.5)	28 (87.5)	0.640
	>1 year	120 (31.3)	19 (15.8)	101 (84.2)	
Oral health-related variables					
Brushing	Yes	344 (89.8)	95 (27.6)	249 (72.4)	0.793
	No	39 (10.2)	10 (25.6)	29 (74.4)	
Frequency of brushing	No or irregular	163 (42.6)	36 (22.1)	127 (77.9)	0.044
	≥1 times/ day	220 (57.4)	69 (31.4)	151 (68.6)	
Who brushes?	Child alone	157 (41)	40 (25.5)	117 (74.5)	0.416
	With help	187 (48.8)	55 (29.4)	132 (70.6)	
Age started brushing	≤3 years	157 (41)	49 (31.2)	108 (68.8)	0.478
	>3 years	142 (37.1)	39 (27.5)	103 (72.5)	
Plaque Index score	Low	114 (29.8)	43 (37.7)	71 (62.3)	0.004
	High	269 (70.2)	62 (23)	207 (77)	
Oral Hygiene Index score	Poor	109 (28.5)	13 (11.9)	96 (88.1)	<0.0001
	Fair	198 (51.7)	52 (26.3)	146 (73.7)	
	Good	76 (19.8)	40 (52.6)	36 (47.4)	

ECC: early childhood caries.

To control for covariant factors, factors with p-value < 0.1 were included in multiple logistic regression. Five significant factors were identified: preschool area, father’s occupation, previous dental visits, history of nocturnal feeding, and oral hygiene status (Table 4). Children from schools in northern Riyadh were less likely to have ECC (OR: 0.203; 95% CI: 0.082–0.503) than children from preschools in other areas. Children of fathers in professional jobs were less likely to have ECC (OR: 0.472; 95% CI: 0.256–0.871) than children of fathers in administrative jobs. Children with nocturnal feeding history were more likely to have ECC (OR: 2.281; CI: 1.143–4.553). Additionally, children who had visited a dentist were more likely to have ECC (OR: 3.050; 95% CI: 1.587–5.862) than those with no visits. Children with poor oral hygiene were much more likely to have ECC than children with good or fair oral hygiene (OR: 5.523; 95% CI: 2.269–13.441).

**Table 4:** Factors associated with early childhood caries in multiple logistic regression analysis.

Factor		Sig	OR	95% CI	
				Lower	Upper
Preschool area	Central	ref			
	East	0.366	0.606	0.204	1.796
	North	**0.001**	0.203	0.082	0.503
	South	0.163	0.556	0.244	1.269
	West	0.560	0.722	0.242	2.158
Father’s occupation	Administrative	ref			
	Professional	**0.016**	0.472	0.256	0.871
Birth order of child	Last	ref			
	First	0.396	0.728	0.349	1.517
	Middle	0.079	0.545	0.277	1.074
Daily feeding frequency (bottle/breast milk)	≥7/day	ref			
	<7/day	0.129	0.621	0.335	1.149
Nocturnal feeding (bottle/breast milk)	No	ref			
	Yes	**0.019**	2.281	1.143	4.553
Sugary drinks in bottle (night)	No	ref			
	Yes	0.406	1.309	0.693	2.474
Water in bottle (day/night)	No	ref			
	Yes	0.080	0.555	0.287	1.073
Consumption of sweets and soft drinks between meals	>1/day	ref			
	No	0.087	0.309	0.080	1.188
	Not daily	0.455	0.656	0.218	1.980
	1/day	0.159	0.418	0.124	1.408
History of dental visits	No	ref			
	Yes	**0.001**	3.050	1.587	5.862
Frequency of brushing	≥1	ref			
	No/irregular	0.647	1.158	0.618	2.171
Plaque Index score	High	ref			
	Low	0.055	0.520	0.267	1.013
Oral Hygiene Index score	Good	ref			
	Poor	**0.000**	5.523	2.269	13.441
	Fair	**0.000**	3.420	1.721	6.797
	Constant	0.067	4.728		

CI: confidence interval, OR: odds ratio.

## 4 Discussion

The present findings demonstrate that ECC continues to affect many Saudi preschool children and is related to socioeconomic factors as well as feeding habits and oral hygiene status. Moreover, our results suggest that most children visit a dentist only after caries has already developed.

### 4.1 ECC prevalence

In this study, the prevalence of ECC remained high in 36–71-month-old Saudi children in Riyadh. This highlights a persistent burden of ECC in preschool children, and is consistent with findings from previous studies in Riyadh using similar diagnostic criteria for dental caries. In 2008, Wyne reported a prevalence of 74.8% and dmft of 6.1 among preschoolers in Riyadh. Later, Al-Meedani and Al-Dlaigan (2016) reported a prevalence of 69% and dmft of 3.4, indicating an improvement, particularly in ECC severity in Riyadh preschoolers. The present study found a higher ECC prevalence (72.6%) and a slight increase in dmft score (4.13). These changes could be attributed to changes in ECC-associated risk factors over time. However, ECC prevalence remains high. Several countries have reported lower ECC prevalence than in Saudi Arabia, namely, Nigeria, India, China, Sudan, Serbia, and Ecuador (Acuña et al., 2019, Elidrissi and Naidoo, 2016, Folayan et al., 2015, Igic et al., 2018, Prabhu et al., 2014, Zeng et al., 2018). We hope that re-identifying ECC risk factors in Saudi preschoolers will help to reduce ECC prevalence in these children by directing the preventive measures.

### 4.2 Sociodemographic and socioeconomic factors

These findings demonstrated that significantly more caries-free children were from preschools in northern Riyadh, similar to previous study findings (Alhabdan et al., 2018). This may be because residents of northern Riyadh usually have a higher economic status than those in other areas. Additionally, schools in northern Riyadh are considered of higher standard and are more expensive, attracting wealthy people from other regions.

ECC prevalence was also significantly lower in children whose fathers were in professional jobs compared with those in administrative jobs. Fathers in professional jobs are usually more educated and have higher incomes. Moreover, father’s occupation may reflect the family income, as fathers are the main providers in Saudi cultural norms (Achoui, 2006). Monthly parental income and low socioeconomic status affect the prevalence of ECC among preschoolers in other populations (Chen et al., 2019, Correa-Faria et al., 2013, Pierce et al., 2019).

In their caries-prediction model, Al Ghanim et al. (1998) did not identify socioeconomic factors as caries risk factors. However, in the present study, preschool area and father’s occupation were significant in the multivariate analysis, indicating their effect on ECC prevalence among Saudi preschoolers. Predictive factors for ECC change with time as socioeconomic dynamics change.

### 4.3 Infant feeding history and current dietary habits

Most children in this study were fed at night, and the frequency and feeding time (day/night) had a greater effect on ECC than feeding period and weaning age. Nocturnal milk feeding and sugary drink consumption in the bottle at night were significantly associated with ECC. However, nocturnal milk feeding was the only factor that remained significant in the regression analysis. Other studies have also reported that nighttime feeding (both breast- and bottle-feeding) after 12 months of age increased the likelihood of ECC (Kubota et al., 2020). Breast-feeding during the first year of life is strongly encouraged (Tham et al., 2015). However, if it is carried out at night or on-demand, with an associated high sugary diet and late introduction of brushing, it can contribute toward high ECC (Branger et al., 2019). The consumption of sweetened drinks is common in Saudi children (Ahmed and Salih, 2019). Nighttime consumption of these drinks was associated with ECC in the present study. During sleep, salivary flow reduces markedly, reducing the self-cleaning effect and buffering capacity of saliva in the oral cavity and shifting the balance toward demineralization rather than remineralization (Nauntofte et al., 2003, Weber-Gasparoni et al., 2007).

In the present study, ECC prevalence was lower in children who drank water in their bottles during infancy. This may be because of the washing effect of water or because of the fluoride content of bottled water, which is consumed by most Saudis (Al-Zahrani et al., 2017). In addition, bottled water is ideal for other uses, such as infant formula preparation (Aleissa et al., 2011).

We found that ECC prevalence increased significantly as the consumption of sweets and sugared drinks between meals increased. This is consistent with previous reports of an association between prolonged high sugar consumption between meals and ECC in children (Folayan et al., 2015, Nunes et al., 2012).

### 4.4 Dental history and oral hygiene habits

Our findings showed that children who had visited a dentist had significantly higher ECC prevalence than those who had not visited a dentist. This could reflect the fact that most children in Riyadh visit the dentist for an existing dental problem (Al-Shalan et al., 2002). A 2016 study showed that only 27.3% of 1–8-year-olds from Riyadh visited the dentist for regular checkups (Murshid, 2016). This may reflect the common belief among parents that children do not need to be taken to the dentist unless they have a dental problem (Al-Shalan et al., 2002). Many studies on other populations also report higher ECC among children who had visited a dentist (Fan et al., 2016, Nobile et al., 2014, Nunn et al., 2009, Schroth et al., 2010).

The prevalence of ECC was higher in children with no/irregular brushing, in accordance with results from a meta-analysis (Kumar et al., 2016). Children with poor oral hygiene and high PI had significantly more ECC, in accordance with previous similar work (Kanasi et al., 2010). Poor oral hygiene was identified as a strong risk factor for ECC in the present study. Many studies on different populations concur that ECC is usually associated with unsatisfactory oral hygiene (Correa-Faria et al., 2013, Hoffmeister et al., 2016).

Although the present findings are important, the results must be interpreted in light of the study limitations. Most of the questionnaire data depended on parents’ recall of events. Potential bias was reduced by validating the questionnaire and by conducting a pilot study. The sample comprised children enrolled in preschools, so the results may not be generalizable to children of the same age group not enrolled in preschools. Nonetheless, these findings could help to develop better prevention strategies that focus on high-risk children.

## 5 Conclusions

The prevalence and severity of ECC among preschool children remains high (72.6%), with a mean dmft score of 4.13 (±3.99) and a mean dmfs score of 7.0 (±9.1). High socioeconomic status reduces the risk of ECC. Nocturnal feeding and poor oral hygiene are high-risk factors for ECC among Saudi preschool children in Riyadh.

## CRediT authorship contribution statement

**Lujane K. AlMarshad:** Conceptualization, Methodology, Investigation, Resources, Writing - original draft. **Amjad H. Wyne:** Conceptualization, Methodology, Writing - review & editing. **Asma M. AlJobair:** Conceptualization, Methodology, Formal analysis, Writing - original draft, Supervision.

## Declaration of Competing Interest

The authors declare that they have no known competing financial interests or personal relationships that could have appeared to influence the work reported in this paper.
